# Bibliographical Notices

**Published:** 1849-08

**Authors:** 


### BIBLIOGRAPHICAL NOTICES.

*Cyclopædia of Anatomy and Physiology.* Edited by ROBERT B. TODD, M. D., F. R. S., &c., &c. 8vo. Parts XXXIV. and XXXV. January and March, 1849.

*Cours de Physiologie fait à la Faculté de Médecine de Paris.* Par P. BÉRARD, Professeur de Physiologie a la Faculté de Médecine de Paris, &c., &c. 8vo. 9 livraisons. pp. 744. Paris, 1848.

*Handwörterbuch der Physiologie mit Rücksicht auf physiologische Pathologie,* u. s. w. herausgegeben von DR. RUDOLPH WAGNER, Professor in Göttingen. 8vo. 18, 19, 20 Lieferungen. Braunschweig, 1848–9.

*Lehrbuch der Physiologie des Menschen für Aerzte und Studirende.* Von DR. AUGUST FRIEDRICH GUNTHER. 1 Band. 8vo. Enthaltend die allgemeine Physiologie. S. 660 Leipzig, 1845. 2 Band. 1. Abtheilung. Enthaltend die specielle Physiologie. S. 304. Leipzig, 1848.

*Lehrbuch der Physiologie für Studirende und Aerzte.* Von D. ARNOLD ADOLPH BERTHOLD, Königl. Hannoverschen Hofrathe, u. s. w. Erster Theil, enthaltend die allgemeine Physiologie. 8vo. S. 382. Zweiter Theil, enthaltend die beersonde Physiologie. S. 640. Göttingen, 1848.

*The Chemistry of Vegetable and Animal Physiology.* By G. J. MULDER, Professor of Chemistry in the University of Utrecht. Translated by DR. P. F. H. FROMBERG, with Introduction and Notes by JAMES F. W. JOHNSTON, F. R. S. L. & E. With twenty illustrations, colored and plain. 8vo. pp. 850. Edinburgh, 1849.

It is not long since we took occasion to bring to the notice of our readers the American reprint of the "Handbook of Physiology," by Messrs. Kirkes and Paget, the primary object of which—as stated by its authors—was "to give such an account of the facts and generally admitted principles of physiology as may be conveniently consulted by any engaged in the study of the science: and more especially such an one [a one] as the student may most advantageously use during his attendance upon lectures, and in preparing for examinations."

With similar objects have two of the works before us—those of Messrs. Gunther and Berthold—been undertaken, and they are calculated to serve the same purpose with the German student, that the work of Messrs. Kirkes and Paget serves with us, and wherever the English language is the vernacular. There is this manifest difference, however, between the English and the German authors, that whilst the former—owing to their acquaintance with the German language—have been enabled to obtain the information, which they cite, directly from the sources; the latter, owing to their want of knowledge of the English language, have derived their scanty knowledge of what has been done by the Anglo-Saxon from translations only. Hence the work of Messrs. Kirkes and Paget is more cosmopolitan in its character.

The *Livraisons* of the *Cours de Physiologie* constitute but one volume of the "Course." Five of the nine are occupied wholly with prolegomena; comprising an inquiry into life; the sources of our knowledge in Physiology; its utility; a parallel between living and brute bodies, and between animals and vegetables; the organization of animals; the humours,; functions; a comparison of animals amongst themselves; and of man and the different races of men.

The seventh, eighth, and ninth *livraisons* are occupied with digestion, the last of them ending—and with it the first volume—with insalivation. From this, the probable extent of the work may be judged of. It is exceedingly diffuse: topics are discussed and details given, which it must be impossible for any student to carry away with him; and on the whole we rise from the examination of the lectures, thus far published, with the firm conviction, that for real utility, the less full—less *complete*, if the epithet be preferred—courses on physiology given in this country are—to say the least of them—*as* beneficial to the student as the elaborate and involved course delivered in the Ecole de Médecine of Paris. It must be admitted to be a fault in the lecturer on any subject to attempt to exhaust it. It is a grievous error, we think, to load it with matters that are of doubtful relevancy, and cannot possibly be retained by the hearer; and whilst, therefore, we would disapprove of the material before us having been delivered as lectures, we can still recommend that it should be studied at home; and are thankful to M. Bérard for having afforded us a useful book of reference on many biological topics for the shelves of the library.

The remainder of the works whose titles are at the head of this article are more adapted for the deep and thorough investigator of Biology. The Cyclopædias of Todd and Wagner have been extolled already in the pages of this journal, and the extensive use made of them by every well informed writer sufficiently attests their estimated value. Each article is assigned to some co-laborer who has made the subject a special department of study, and the very assignment has in many cases given occasion to renewed investigation by experiment and otherwise; and, accordingly, the articles in both works may be regarded as embodiments of the existing state of knowledge on the subjects on which they treat.

It may be sufficient for us, after these merited encomiums, to enumerate the subjects and their respective and respected authors in the parts of both works recently received, and now before us.

Parts 34 and 35 of the Cyclopædia of Anatomy and Physiology, contain the conclusion of an article on Secretion by Dr. Carpenter; Semen, by Drs. Wagner and Leuckardt; Sensation and Sensibility, by Dr. Todd; Serous and Synovial Membranes, by Dr. Brinton; Sesamoid Bones, by Mr. S. R. Pittard; Seventh Pair of Nerves, by Dr. Brinton; Shell, by Dr. Carpenter; Shoulder Joint, by Dr. McDowel and Mr. Adams; Sixth pair of Nerves, by Dr. Brinton; Skeleton, by Mr. Maclise; and Sleep, by Dr. Carpenter.

The 18th, 19th, and 20th parts of the "Dictionary of Physiology" of Wagner contain:—Synovia, by Prof. F. M. Frerichs; Temperament, Physiognomy, and Cranioscopy, by Dr. E. Harless; Secretion of Tears, by Prof. Frerichs; Transudation and Endosmose, by Dr. Vierordt; Digestion, by Prof. Frerichs; Physiology in its application to Opthalmology, by Prof. Ruete; Disease, by Prof. Hasse; Physiology in its application to Surgery, by Dr. Kohlrausch; Waking, Sleep, Dreaming, &c., by Prof. Purkinje.

Lastly:—the work of Mulder comes to us with all the authority of a master in chemistry, if not in biology:—the renowned rival of his brother in science at Giessen :—" brother in science," but by no means in feeling on the interesting matters on which both are engaged. It has been painful, indeed, and injurious to the great cause of whieh they are ornaments to witness the controversial spirit they have exhibited towards each other.

Mulder's work is rich in information to the chemist and physiologist, and is eminently worthy a place in the library of the advanced student.

It is pleasing to observe the absorbing interest felt at this day in the investigation of physiological subjects. Physiology and its twin sister pathology, ought to be studied from the first entrance of the student on the threshold of medical science. Careful observation of phenomena, and a rigid deduction of laws from a consideration of phenomena, characterize the man of science, and distinguish him from the empiric—we use the word in a good sense, as it was employed of old ;—and there are no subjects of science the study of which more signally discipline the mind than the two in question. It is, indeed, to a want of proper mental discipline, of an observance of the strict rules of logic, and to deficiency in physiological and pathological knowledge, that we must ascribe the hasty inferences from isolated facts, in place of sound generalizations formed from a wide and extended view of all the ramifications and bearings of a subject, that we owe those hypothetical assumptions, and absurd modes of management founded on them, that for a while agitate many of the profession, and more of the laity, then sink into insignificance and oblivion, but still add to the numerous examples which the world are disposed to bring forward of the uncertainties of medical science.

When wide spreading sickness assails a community, any proposition that may seem to have the slightest authority in its favor is clutched with avidity ; and, too often, the mercenary and the designing, for unworthy purposes, and the enthusiastic but deluded with the best of motives, appear before the public with propositions, many of which are ridiculous, others plausible but unsupported by observation ; and in this manner more real injury is inflicted on the profession, than could be by its most prejudiced and hostile denouncers.

Witness the sensation recently created by the idle statement in regard to the prevalence of ozone as a cause of cholera. That it may have been indicated in some places, in a greater amount than usual, when cholera prevailed, we do not mean to contest; but when a wide view is taken, we find, that where the disease was most disastrous, reagents did not in the least indicate its presence. Yet, to cap the climax of absurdity, pills of sulphur and charcoal have been proposed by a member of the profession as neutralizers of an atmospheric condition, of which we know scarcely anything; which was not shown to exist in undue amount, and, as we have said, has been certainly proved to be non-existent where the mortality from cholera was excessive: and even were its presence demonstrated, is it shown that either sulphur or charcoal could diminish its amount? and were it so shown, what could be the beneficial effect of a few grains of either swallowed by a patient? We notice the absurdity only because the uninformed, professional and unprofessional, have believed, that there was some foundation for the idle advertisements of ignorant, and we fear, designing persons, against the reception of whose assertions, proper habits of observation and reflection would have effectually steeled them.

Similar remarks may be made on the notion, that an absence of electricity in the air is a cause of cholera. That there has been such apparent absence in many cases where cholera has prevailed is unquestionable. The unfrequency of thunder in this city for the last few months has been a common subject of remark, but it is as unquestionable, that such unfrequency has existed where no cholera prevailed; and, consequently, something more is necessary to cause it: and, moreover, in many places thunder storms have been common, whilst the disease was sweeping off unusual numbers. We are therefore justified, we think, from an examination of the occurrence of the disease on a large scale, in according with a recent writer, that the conditions, which favor the development of cholera, are comparatively independent of temperature, barometrical changes, and atmospheric vicissitudes. Generally, its inflictions have been most severe during the warmer months; but in Moscow it raged during the rigor of a Russian winter, and passed away on the return of mild weather. The history of the recent visitation in New Orleans is also full of interest in this relation.

But we do not intend to enter into the various anomalous positions that have been assumed in regard to this singular pestilence.

Hereafter we may take up the subject, and endeavor to demonstrate, that a number of "vulgar errors" prevail even amongst the profession, connected with the influence of atmospheric temperature, particular kinds of diet, &c., &c. A more fitting time for such a discussion will arrive when existing agitations have passed away; and a cool review can be made of the whole subject. The object with which the allusion to cholera has been made by us was to deplore the crude deductions unadvisedly put before the profession and the public in periods of agitation and alarm, and to express our conviction, that the mind properly disciplined in the kind of reasoning which physiology so eminently demands and fosters, would at once discover the fallacy of such deductions: and the same kind of mental training would make the philanthropist pause before he committed himself and his profession by their too hasty promulgation.

---

*Speeches of Defendants' Counsel, and the Charge of Judge Burnside, in the case of Hinchman vs. Ritchie, et. al.* Philadelphia, 1849. pp. 176.

This pamphlet contains a full and accurate report of the arguments adduced by counsel, to defend a body of respectable citizens from the serious charge of a conspiracy to deprive a man of his liberty and property under the plea of insanity. The parties arrayed against each other in this extraordinary cause, were closely allied by the ties of nature—the son appears against his mother, the husband against his wife, the brother against his sister, the paternal relatives of the prosecutor against his maternal relatives, and the mutual friends of each testify upon opposite sides. The prosecutor alleges that while in the exercise of his lawful business, coming to Philadelphia from the country, to dispose of the produce of his farm, he was seized in a clandestine manner by the defendants, who had arranged a plan beforehand for capturing him, and conveyed, without his consent, in his own wagon to an insane asylum, and there incarcerated without just cause for a period of several months. That shortly after his confinement a commission of lunacy was taken out, and his property passed into the hands of others, to his great loss and detriment. After being discharged from the institution as a sane man, he appeals to the law for redress, and brings suit for damages against the parties concerned in placing or keeping him there, including all his nearest relations, several intimate friends, the physician who gave the certificate, the medical attendant of the hospital who had charge of him after his admission, and his family physician, who advised him to yield to the wishes of his relatives, and was present when he was taken to the institution : in all, fifteen persons.

To sustain the charge of conspiracy, the plaintiff first attempted to prove his sanity by a large number of witnesses, who were his neighbors or intimate friends. Some sixty or seventy persons testified that they had never observed anything strange or crazy in the plaintiff's conduct—some even declared that he was a remarkably shrewd, business man, smart and capable, &c., while it was agreed on all sides that he was not deficient in business capacity.

On the other side it was alleged that the plaintiff was subject to paroxysms of insanity; that there was on his part a " prolonged departure, without adequate or external cause, from the state of feeling and modes of thinking which were usual to him when in health," and a number of witnesses, some of whom were near relatives and intimate friends, were brought forward to testify to facts and circumstances which had convinced them that his mind was diseased. This testimony extended through a period of seven or eight years, and had reference to many circumstances in his private and domestic life, which induced his near relatives to think it advisable to place him under medical treatment in an insane asylum.

In doing so they alleged that they were solely influenced by motives of sympathy and humanity, and that they had applied to the persons now included with themselves in the charge of a conspiracy, for their friendly aid in effecting this object. The regular forms of admission were complied with : and the parties charged declared that they had no interest, pecuniary or otherwise, in the confinement of the plaintiff, farther than the restoration of his health, and the security of his remaining property for the benefit of his family.

In presenting this brief view of the position of the opposing parties, we only desire to state the points at issue so far as the medical bearings of the question are concerned. Upon the legal points, and the various other matters with which the case is complicated, we do not feel prepared to comment.

The question of the insanity of a man, in which is involved the right to arrest and confine him, and to dispossess him of his property, is one of very grave import. It is one, the decision of which by the practice of this State, has been vested in his medical attendant. The certificate of the physician is here the groundwork of the proceedings whereby he can be thus confined; his signature is, in fact, equivalent to the commitment of the judge or magistrate in a criminal case. The responsibility here assumed by the physician is therefore of high importance, and should be exercised only after the most careful scrutiny into the history and appearances of the patient presented to his examination. So deep and mysterious are the workings of the human mind, and so disjointed may the social and domestic relations of life become, through the indulgence of improper passions, and the want of self-control, that nothing short of a conviction of a man's insanity, founded on the most satisfactory proof, should induce the physician to act as his committing magistrate.

Especially is this caution necessary in cases of partial insanity, where the individual himself denies that his mind is diseased, and where his ordinary course of conduct does not betray the existence of the malady to those with whom he is in daily intercourse, and where too he protests against being restrained of his liberty. Not that such persons do not require restraint, and that the exercise of a sound discretion on the part of the physician may not save them from violent and even hopeless mania; but in effecting it great circumspection should be observed, deception and stratagem should be if possible avoided, and the individual be approached with that regard for his rights and feelings, and that sympathy for his welfare, which might, if possible, secure his cooperation, rather than excite his enmity and opposition. The records of institutions for the insane would present many examples of individuals thus diseased, who have themselves been convinced of the necessity of being subjected to the influences which these excellent establishments exert, and who have voluntarily and even cheerfully entered them. The humane and enlightened spirit in which insane asylums are now generally conducted, have, in fact, robbed them of the terrors once attached to them, and have rendered them pleasant retreats from the excitement and turmoil of the world, rather than the abodes of cruelty and misery. In the case before us, the ground was boldly assumed by the counsel for the plaintiff, that the exercise of the right to arrest and confine an insane person on the certificate of a physician, as this proceeding was not upon oath or affirmation, was contrary to the bill of rights, and a violation of the Constitution of Pennsylvania. But Judge Burnside, in charging the jury, negatived this proposition, and declared that the rule which had been practiced for the admission of insane patients into the Pennsylvania Hospital, half a century before the adoption of the Constitution, and which had been pursued by the Frankford Asylum since its organization, without being questioned by the framers of the Constitution, or by the convention for its amendment in 1838, gave full authority to this proceeding; provided it was proved that the man was insane. "The right to restrain an insane person of his liberty is found," as expressed by Chief Justice Shaw of Massachusetts, "in the great law of humanity." The duties and responsibilities of physicians continue, therefore, undisturbed by the decision of this case.

The next question is, how far medical men may render themselves liable to prosecution or annoyance, by the exercise of the prerogative which is thus imposed upon them? Heretofore, no apprehension seems to have been felt upon this point, and it is a remarkable fact, that during the long period in which the certificate of the physician has been considered as the warrant for the arrest and confinement of insane persons, no instance has occurred in this State of a prosecution against the medical attendant, except in the case now under review. In this case four physicians were included in the indictment for conspiracy, viz: Dr. Kite, who signed the certificate of admission; Dr. Griscom, the family physician of the plaintiff, who was present when he was taken, and who advised him to accompany the friends who had volunteered to convey him to the asylum; Dr. Evans, the attending physician of the asylum, under whose authority and advice he was restrained in the institution, and Dr. Worthington, the resident physician. Whether all these gentlemen were thus arraigned for the purpose of excluding their testimony upon the main point at issue, viz. the insanity of the plaintiff, or whether from the desire of convicting them as parties in a conspiracy, we shall not undertake to determine. Had they all been in the position of defendants during the whole proceedings, it is evident that the most important link in the testimony upon which the accused parties relied would have been excluded, and their chances of conviction thereby greatly increased.

Application was made, however, in the early part of the proceedings, for the release of the physicians, which resulted in the discharge of Dr. Evans, who became an important witness in the case.

The three other physicians were still held accountable, and one of them, Dr. Kite, was actually convicted as a party in the conspiracy, and is now subject to the penalty imposed, unless the decision should be reversed by a higher court. Dr. Griscom, though not convicted, was subjected to the anxieties and annoyance of a protracted and exciting trial, and was held up to public censure by heated partizans, for having acted the part of a friend and counsellor to a man in whose welfare he had long been interested; and Dr. Worthington, who simply acted in the position of resident physician of the asylum, was subjected to like treatment, though he also escaped conviction.

Well might the learned judge remark, in charging the jury upon the cases of the medical defendants, that the conviction of either of these gentlemen "would deter physicians from giving these certificates, and have an injurious effect upon society." If physicians, acting in the capacity of advisers to those who apply to them for professional aid, oftentimes without pecuniary reward, and without their own seeking, are to be arraigned before courts of justice as malicious conspirators against the rights and interests of their patients, without the shadow of proof of evil intention or bad motives, and when in the legitimate exercise of those responsibilities which the customs of society and the laws of humanity have imposed upon them, they may indeed hesitate to subject themselves to this new species of martyrdom, in addition to those troubles which the exercise of their calling necessarily brings with it.

The true position of medical men in this relation, did not escape the notice of the counsel for the defendants. George Griscom, Esq., the attorney for Dr. John D. Griscom, has thus stated it.

"*Sixth.*—That the *professional advice*, or opinion of a physician, given to any one to whom he is called to give it, honestly and truly, with intention to benefit the health of the person advised, without any other action on the physician's part (excluding the idea of malpractice, of course,) cannot, in any case, subject the physician to an action or damages."

To this Judge Burnside replies.

"*Answer.*—Undoubtedly, where a physician gives a certificate honestly and *bona fide,* he ought not to be subjected to damages."

In relation to the case of Dr. Kite an exception is made by the Judge in the following words:

"As for Dr. Kite if he were not in the original conspiracy, and gave a conscientious certificate, I would not hold him responsible ; nor do I see the legal principle upon which he can be convicted. His conviction would deter physicians from giving these certificates, and have an unhappy effect on society. But really did he give a corrupt certificate ? If the jury really believe that Dr. Kite did give a corrupt certificate it would materially alter the case, and there is one thing that makes against him : he stated, according to the evidence, that he had not seen the patient in four months ; this I think is the strongest feature in his case. The rulers of the institution do not require that the certificate should state when the examination, upon which it was based, was made. The British statute requires that the certificate should state when the examination was made, which must not have been made more than seven days previous to the date of the certificate ; and that two physicians must be present at the examination. If the managers of this institution would take my advice, they would adopt the provision of the British statute, and require that all examinations should be personal, and it should be stated on the certificate when they were made. However, gentlemen, it is for you to judge of Dr. Kite. For the mere giving of the certificate I would not find him guilty, unless you believe he certified falsely. If he acted conscientiously, I think he ought to be discharged."

This informality in the medical certificate is certainly to be regretted, and cannot be justified as a safe rule of action. The necessity of a personal examination at the time, or immediately preceding the signing of a paper from which such important results flow, is obvious, and might not, we think, be inappropriately required as a condition of its validity. But we cannot understand, how an inadvertence on the part of the physician in this matter, can justly subject him to an action for maliciously endeavouring to deprive a man of his liberty and property, when it is not alleged that he had any pecuniary interest to subserve, or any malice to gratify, by such act.

Another feature in this case which is interesting to medical men, is the popular view of insanity, as revealed in the progress of this trial, and attested by the verdict of the jury, as distinguished from the medical or scientific view of the question, as sustained by the best medical observers and writers upon this disease.

The people and the profession are here at issue. The popular ideas of the nature of insanity, are so loose and indefinite, that in doubtful cases no positive information can be derived as to the state of a man's mind, from the testimony even of honest and intelligent witnesses. Capability in attending to the ordinary business of life, the power of maintaining a rational conversation upon a given topic, or the absence of violent fits of insanity before transient observers, are all regarded as proof positive of a man's sanity. Monomania, moral insanity, and various other aberrations of intellect, which are familiar to medical men, are not admitted by the public, who in this matter undertake to form opinions with as much confidence as the most acute physician. In the case of Hinchman, for instance, popular opinion attached much more importance to the testimony of his neighbors and friends, than to that of Dr. Evans, the only physician who, from his position, was enabled to give a decided opinion in the case ; whereas, intelligent medical men would regard the opinion of Dr. Evans, stated as it was in a clear and lucid manner, and based upon sound medical views, of more value than that of a score of casual observers, who had no knowledge of the nature and peculiarties of this form of disease.

In the volume before us we find a full discussion of the question of insanity as affecting the intellect, feelings, and passions, in its relations to the case in hand.

The counsel for the defence appear to have studied this branch of their case with great care, and have presented an elaborate and able argument for the generally received medical doctrines upon this subject. The speech of Charles Gibbons, Esq., is especially full upon this point, and is replete with much valuable information, drawn from a variety of sources. We could wish that his brethren of the bar, and the intelligent masses generally, would read and ponder this address. As the matter now stands, it is essential for the ends of justice, that the public should be informed upon the nature and phenomena of insanity; and that they should be instructed in the various phases which it is constantly assuming.

In them the right to decide upon a man's mental condition, in cases involving life, reputation and property, is vested. Courts expound the law, and juries render the verdicts. They are often called upon to determine, where moral accountability ceases, and insanity begins ; what acts are the result of criminal and vicious propensities under the control of the individual, and under what circumstances the same acts are to be regarded as the offspring of an insane and uncontrollable impulse. These are serious questions which are continually presenting themselves in courts of justice, and in the decision of which momentous consequences are involved. Murder, arson, theft, and other grave crimes, are occasionally committed under circumstances in which the guilt of the perpetrator may be fairly questioned ; while in other instances the plea of insanity is set up without just cause, and as a means of shielding the criminal from the punishment which his crime merits. Insanity in its relations to penal law, is, in fact, one of the most perplexing and abstruse problems, which can occupy the minds of jurists and physicians. It is daily forcing itself upon public attention in a variety of forms, and is demanding a thorough examination. As an element in the administration of the law, modifying its penalties, and influencing its decisions, it cannot be too closely studied; but whether in the present state of our knowlege, settled principles could be fixed for the guidance of courts and juries, is at least doubtful. In our view the only tribunal, competent to decide upon these difficult questions, should be composed of medical men, whose positions familiarized them with insanity in all its protean forms, and who have made it an object of special study.

This class of physicians are to be found in most of the states of the Union, and a more intelligent and highly educated body of men than those who now manage the numerous insane hospitals of the country, do not exist in the ranks of any profession.

Let the law invest these gentlemen with powers which are now in the hands of judges and juries, and secure their aid and counsel in cases where insanity complicates its decisions, and one source of difficulty would be removed. In some of the states this course has been partially adopted. In Massachussets, for instance, a medical commission inspects the prisons, and decides upon the state of mind of such convicts as are considered of doubtful sanity, and their report determines the continued incarceration of the subject, or his removal to an insane asylum.

If the same rule were extended to untried prisoners before conviction, and if conscientious and practiced physicians became the arbiters, under proper legal restrictions, instead of juries, we believe that society would be better protected, while individual rights would be rendered more secure.

The case of Hinchman, which has excited such extraordinary public interest, may have the effect of bringing about a renewed investigation on the part of competent persons, of the relations of insanity to the administration of the law, which shall ultimately lead to important changes in this branch of legal practice. Already the subject of the duties and responsibilities of physicians in this respect, is beginning to demand their serious attention, and at the last meeting of the National Medical Association at Boston, a committee was appointed to examine and report upon it. Upon this committee are several gentlemen widely known for their labors in the cause of the insane, and for the zeal and ability with which they have cultivated this department of medicine. From their united labors the profession and the public may anticipate a report of great value, which may serve as a guide for future action. Viewed in this light, the "Hinchman case" may not be without its uses; and when the private griefs and domestic calamities to which it has given rise are forgotten, it may be referred to as an important public trial which has called forth the discussion of great principles, and aided in the establishment of sound views upon an obscure and difficult subject, intimately connected with the welfare and happiness of the commonwealth.

---

*Report on the Cholera in Paris. Published by authority of the French Government, translated from the original, and printed by recommendation of the Board of Health, and the Academy of Medicine, of the City of New York.* New York: Samuel S. and William Wood, 1849.

The above report, one of the products of the international exchange, effected by M. Vattemare, was made by the direction of the French Government in 1832, immediately after the cessation of the cholera in the metropolis. The commission to whom was intrusted the duty of preparing the report, consisted of men known throughout Europe and this country, and belonging to various learned and scientific bodies, and holding important official stations. Its republication at this juncture is an inestimable favor to all corporations and boards of health.

It will be seen that the report was prepared after the subsidence of the epidemic, when the violence of the disease had abated, and when no longer under the influence of *immediate* impressions, and more sure in their judgments, they were enabled to recur to what had passed, and direct attention to the examination of circumstances and localities, and to obtain such a precise account of the character and progress of the pestilence, as would serve, should it suddenly re-appear, to enlighten the present by the experience of the past; or to instruct for the future, should the disease make its appearance only at long intervals.

It will be seen also, that they have avoided all abstract theories, and confined their attention to the close examination of the circumstances under which the disease first made its appearance, and which accompanied its progress. Having no particular doctrine of *contagion* or *non-contagion* to support, they have not ventured to offer other than the most certain and legitimate deductions even from the facts observed and recorded by themselves.

Every circumstance bearing upon the subject has been examined with a minuteness and clearness of detail, that we do not remember ever to have seen excelled in any similar report. "The geographical position, and geological formation of the district of the Seine; the number and size of the streams by which it is watered; the extent of woods, or of roads, which diversify and traverse its surface in every direction; the dryness and humidity of the atmosphere; the nature of the winds usually prevailing at different seasons;—each and all of these became subjects of research, with the view of ascertaining how far, and in what manner, their influence was exercised on the pestilence which then raged.

From these subjects, the members of the committee necessarily passed to the consideration of others of equal if not greater importance: in what respect density of population, or the neighborhood of hospitals, slaughter houses, burying-grounds, manufactories, &c., operated on the increase or diminution of the disease; how far it was modified by the ages, habits, pursuits, or trades of individuals; and lastly; what means were employed by the Government and its agents to avert the progress of the cholera."

The following summary of this model report will give our readers some idea both of the extent of the inquiries and the conclusions at which the commission arrived. We would, however, commend the whole paper to the careful study and imitation of all interested in this, at present, all absorbing subject.

1. The cholera appeared at one and the same time in Paris and in the rural communes of the Department; or, to be more positive, within an interval of 48 hours, from the 26th to the 28th of March.

2. In the country, as in the city, its developement, its progress, its periods of abatement or increase (recrudescence,) as well as its duration, have been similar.

3. In the country, as in the city, more women than men have died, but in the country the mortality of the females was one fifth greater than that of males, and comparatively larger than in Paris.

4. In the rural communes, as in the city, the ages that seemed most liable to disease and death, were first infancy, mature age, and senility; the period of human life that suffered least is that between 6 and 20; but in the rural communes, first infancy experienced relatively to other ages a greater loss than in Paris, and adolescence a lesser loss as well as persons advanced in life. Compared to the chances of ordinary mortality, the age between 30 and 40 is that which has presented every where the most unfavorable results.

5. The resistance of nature to the attacks of the disease, has been in a direct ratio to the strength that age offered, excepting however the period from 5 to 10 years.

6. It does not appear that the variations of the atmosphere exercised more influence on the activity or relaxation of the evil, in country than in town.

7. The total population of Paris lost

| | | | | | |
|---|---|---|---|---|---|
| | 18,402 | persons, or | 23.42 | out of | 1000. |
| Of the wards of Saint-Denis, | 2,001 | do. | 21.03 | | do. |
| Of the wards of Sceaux, | 1,385 | do. | 17 62 | | do. |
| Total in the whole Department, | 21,514* | do. | 21.75 | | do. |

And if the rural communes suffered less than the capital, the recrudescence in July proved more fatal in them in proportion to the total loss.

8. The rural communes most exposed to the winds were most

* From the 1st of October, 1832, to the 1st of April, 1833, the number of persons, whose death has been attributed to cholera, was for Paris 714, and for the country 80; giving 22,308 victims, or 23.57 out of 1000 as the deaths by cholera from the time of its invasion in March, 1832.

assailed, but in Paris the central districts and narrowest and best sheltered streets, suffered most severely. Generally in the localities last mentioned, wherever a poor wretched population was crowded in filthy, contracted lodgings, the epidemic multiplied its victims.

9. In the rural wards, as well as in the capital, the cholera seems to have more specially struck at the professions that indicate least comfort, and above all at those which are exercised in the open air.

10. The excesses in which, too often, the working classes of Paris indulge on Sundays seem to have produced one-eighth of augmentation in the number of admissions to the hospitals on the Mondays following.

11. The mortality was less among prisoners than among other classes of the Parisian population.

12. The loss experienced in the hospices, taken as a whole, presents the same proportion, 64 out of 1000, that is presented by the deaths of the inhabitants of Paris of 60 years and upwards.

13. The military fell before the pestilence, both in Paris as in the rest of the Department, in the proportion of 25.66 out of 1000; a proportion which surpasses that of the civic population (21.83.)

14. Lastly, in places infected by putrid emanations, the cholera was neither more extended nor more fatal than in other localities.

